# Women living with HIV face intersectional stigma from infection, domestic violence, and other marginalized identities: a qualitative study in West Bengal, India

**DOI:** 10.1186/s44263-024-00122-w

**Published:** 2025-01-10

**Authors:** Reshmi Mukerji, David Osrin, Jenevieve Mannell

**Affiliations:** Institute for Global Health, University College London, 30 Guilford Street, London, WC1N 1EH UK

**Keywords:** Intersectional stigma, Domestic violence, Women, HIV, India

## Abstract

**Background:**

Women living with HIV bear a disproportionate burden of stigma, especially in countries where gender discrimination is more common. A result is widespread domestic violence against women. This violence is itself stigmatized, but the intersectional stigma of HIV and domestic violence has not been well studied. Our work aimed to fill this research gap by exploring how domestic violence and HIV stigma intersect with other marginalized identities in women’s lives.

**Methods:**

Semi-structured interviews were conducted in Kolkata, India with 31 women living with HIV and 16 key informants to gain an understanding of intersecting stigmas. Interviewees discussed women’s experiences and perceptions of stigma and discrimination around HIV, domestic violence, and other marginalized identities. The coding of data was informed by an intersectional stigma framework. Thematic Network Analysis was used to group themes originating in the data into higher-order themes connecting to a global theme.

**Results:**

The findings presented are a qualitative self-report of violence. The three main themes developed were (1) the intersectional stigma of HIV and domestic violence amplified stigma as a whole, (2) the intersection of multiple stigmas worsens domestic violence, and (3) the stigma of HIV hides domestic violence. Specifically, HIV stigma triggered domestic violence and perpetrators reinforced HIV stigma through verbal abuse. Women with other marginalized identities, such as having daughters or being a widow, experienced substantial violence. Domestic violence stigma was worsened by HIV stigma as women hid the violence for fear of revealing their status. As a result, help-seeking from formal and informal sources decreased, which increased women’s isolation.

**Conclusions:**

The findings shape new understandings of how intersectional stigma of HIV, domestic violence, and marginalized social identities interact to amplify stigma and related violence. Women living with HIV who have multiple marginalized identities should be prioritized for violence reduction interventions.

**Supplementary Information:**

The online version contains supplementary material available at 10.1186/s44263-024-00122-w.

## Background

India has witnessed a feminization of the HIV epidemic. Although there has been a decline in overall prevalence, the proportion of people living with HIV who are women increased from 29% in the 1990s to 46% in 2022 [1, 2]. Domestic violence has been identified as a significant risk factor for HIV acquisition among women in South Asia; a region where high levels of domestic violence are reported [3]. Gender inequality is often a key structural driver of domestic violence [4]. Violence can take many forms including physical violence (beatings), sexual abuse (forced sex), psychological abuse (verbal abuse, threats, humiliation), and financial abuse (dowry demands, restricting access to financial resources) [5]. For women living with HIV, the violence may be driven by a combination of gender discrimination and HIV stigma [6, 7]. Domestic violence is itself stigmatized, with one manifestation of this stigma being reduced help-seeking among survivors [8–10]. One of the most widely recognized theorists on stigma, Goffman, conceptualized stigma as a socially undesirable attribute that can discredit an individual [11]. Others have argued that stigma operates when there is a power differential and is exercised through labeling, stereotyping, marginalizing, excluding, causing a loss of status, and discriminating against the stigmatized individual [12].

Although both people living with HIV and survivors of domestic violence are stigmatized, the stigma of HIV often manifests differently from that of domestic violence. However, when the two stigmas intersect, it can result in greater silence around domestic violence as women living with HIV and experiencing violence often may not get the sympathy accorded to other survivors of violence [7]. A substantial body of literature reports on how domestic violence commences or worsens for women after an HIV diagnosis [13–16], but whether it changes when women have other stigmatized identities or social positions, such as sex worker, widow, or belonging to lower socioeconomic position, is less well understood. The intersectional framework, first developed by Kimberlé Crenshaw in describing the experiences of African American women, has provided a powerful lens through which to examine intersectional stigma [17]. An Intersectionality framework underscores the importance of recognizing the complexity of people’s lives and how interlocking structures of oppression caused by social inequalities shape individual experiences rather than a single marginalized identity [17, 18]. As stigma reproduces and reinforces social differences, understanding the effects of HIV-related stigma is not complete if not accompanied by an understanding of how it strengthens and reproduces existing inequalities, such as those of gender, class, sexuality, and ethnicity [19]. The term intersectional stigma encompasses these ideas as “a concept that has emerged in the literature to characterize the convergence of multiple stigmatized identities within a person or group and to address their effects” [20]. It emerges from the concept of intersectionality which Logie et al. defines as an “interdependent and mutually constitutive relationship between social identities and structural inequities” [6].

Studies from the USA and Canada have used an intersectional stigma framework to show how marginalized identities such as ethnicity, gender, sexual orientation, sex work status, and poverty have intersected with HIV stigma to shape the distinct experiences of women [6, 21, 22]. However, an intersectional framework acknowledges the dynamic nature of social inequalities and marginalized identities, such that they not only change over time but are contextual and often differ with cultural and geographic settings [20, 23]. While it may be crucial to incorporate understandings of ethnicity or sexual orientation for women living with HIV in the USA, such identities have different relevance in a setting such as India, where identities such as caste, class, ethnic background, religion, or widowhood may be salient. In addition, contextual factors such as poverty may further complicate the stigmatization process in this setting, as persons of lower socioeconomic position not only lack financial protection but are considered low class, which risks worsened stigma [24, 25]. There is, therefore, a need to understand how women living with HIV are stigmatized in the Indian context and how these stigmas intersect in the lives of women in a social context where domestic violence is widely accepted as normal.

In this study, we conducted interviews with both women and key informants in order to examine the perceptions and experiences of intersectional stigma of women living with HIV, from the wider population as well as sex workers, in Kolkata, India. This will help to shape understanding of how multiple stigmas combine in women’s lives.

## Methods

### Study location and recruitment

The study was conducted in Kolkata, India in 2020–2021, as a larger project on the health and social effects of intersectional stigma experienced by women living with HIV in India. The methods have been detailed elsewhere [26]. West Bengal is an appropriate setting for studying intersectional stigma because of its geographical location, bordering Nepal and Bangladesh, which makes it a human trafficking zone [27]. The capital, Kolkata, is one the four largest cities of India and the state also attracts migrants from neighboring states for economic reasons. In addition, the state reports high HIV incidence rates [28] and ranks highest in the country for reported cases of domestic violence [29].

COREQ guidelines have been followed for reporting the research process (Additional File 1: COREQ checklist).

We conducted 31 semi-structured face-to-face interviews with women aged over 18 years living with HIV (Table 1) and 16 semi-structured interviews, on a secure video conferencing platform, with key informants in the fields of HIV, medicine, law, and domestic violence support (Table 2). The inclusion of key informants was important to the study as it helped provide contextual information and additional perspectives on multiple stigmas and structural discrimination experienced by women living with HIV.

**Table 1:** Key characteristics of women living with HIV

Women living with HIV	Number (%)
Type	
General population	26 (84)
Sex worker	5 (16)
Age	
Below 35	11 (35)
35 and above	20 (65)
Religion	
Hindu	25 (81)
Muslim	6 (19)
Native language	
Bengali	24 (77)
Hindi	7 (23)
Marital status	
Married/Remarried	13 (42)
Separated/single	9 (29)
Widowed	9 (29)
Partner serostatus	
Seroconcordant	24 (77)
Serodiscordant	2 (7)
Not known/not applicable	5 (16)
Total	31 (100)

**Table 2:** Key informant characteristics

Key informants	Number
HIV physician	3
HIV nurse	1
Counselor/social worker	3
Legal	2
Activist	2
HIV/sex work NGO	2
Domestic violence NGO	1
Public health official	1
Clinical psychologist	1
Total	16

Key informants were recruited through personal contacts and snowball sampling [30]. They were invited by phone or email and sent a consent form for a signed return before the online interview. Two key informants refused to participate, despite initially agreeing to do so, due to time constraints or not wanting to give signed consent. A purposive sample of women living with HIV was recruited through a non-government organization (NGO) providing HIV treatment and outreach support to people living with HIV. The broad inclusion criteria of all women above the age of 18 years living with HIV allowed for as diverse a sample as possible, which would uncover a range of stigmatized identities (such as sex worker, widow, survivor of trafficking) that women might have. Women living with HIV who were below 18 years of age and/or had never received services at the NGO were excluded. Women were invited when they were attending an outpatient clinic, visiting the NGO for other activities, or because they lived on the premises. A counselor or outreach worker (a staff member providing outreach support to beneficiaries) made first contact with participants. If they agreed to interview, they were asked to speak to the researcher, who explained the project in greater detail and followed an informed signed consent process. Considering the sensitivity of the topic, rapport was established through regular visits to the NGO and talking to participants before the interview to make them comfortable with the process. None of the women refused to participate nor withdrew after recruitment. A written summary was maintained after each interview and the potential emergence of themes was noted and discussed among the research team. Saturation was determined through this process when no new information was seen to be emerging from the interviews, at which point recruitment was stopped.

### Data collection and analysis

Interviews with key informants allowed for triangulation of findings from interviews with women living with HIV. Face-to-face interviews with women living with HIV were conducted in a private room at the NGO, with only the interviewer and participant present. All interviews were conducted by a woman doctoral researcher who was a native Bengali speaker with a Master’s in Public Health (RM). She had training in sensitive interviewing and had previous experience conducting qualitative health-related stigma research in a similar setting [31], which led to her interest in this topic. Participants were informed of the researcher’s background as well as the goals of the study. Some demographic information such as age, religion, marital status, number of years living with HIV, and occupation was collected. An interview topic guide was prepared (Additional file 2: Participant interview guide), although the interviewer had the option of exploring additional topics. The topic guide was divided into sections to cover questions on experiences of intersectional stigma and resulting domestic violence and the health consequences of such experiences. Although the interviewer followed this structure generally for all 31 interviews, she had the flexibility to move back and forth between sections. Interviews were audio-recorded using a digital voice recorder (SONY ICD-PX470, Sony Group Corp., Japan). Field notes were prepared to record non-verbal cues and other emotions that participants expressed. After the first two interviews, the researcher went through the recordings and notes and amended the topic guide to include new probes and highlight topics of interest.

A separate topic guide was developed for key informants (Additional file 3: Key informant interview guide). They were asked about their perceptions of stigma and violence experienced by women living with HIV. Although questions were generally the same, minor modifications were made so as to capture each person’s field of work. No demographic or other background information was collected from key informants, but most gave a brief introduction about themselves and their work related to people living with HIV at the beginning of the interview.

**Fig. 1:**
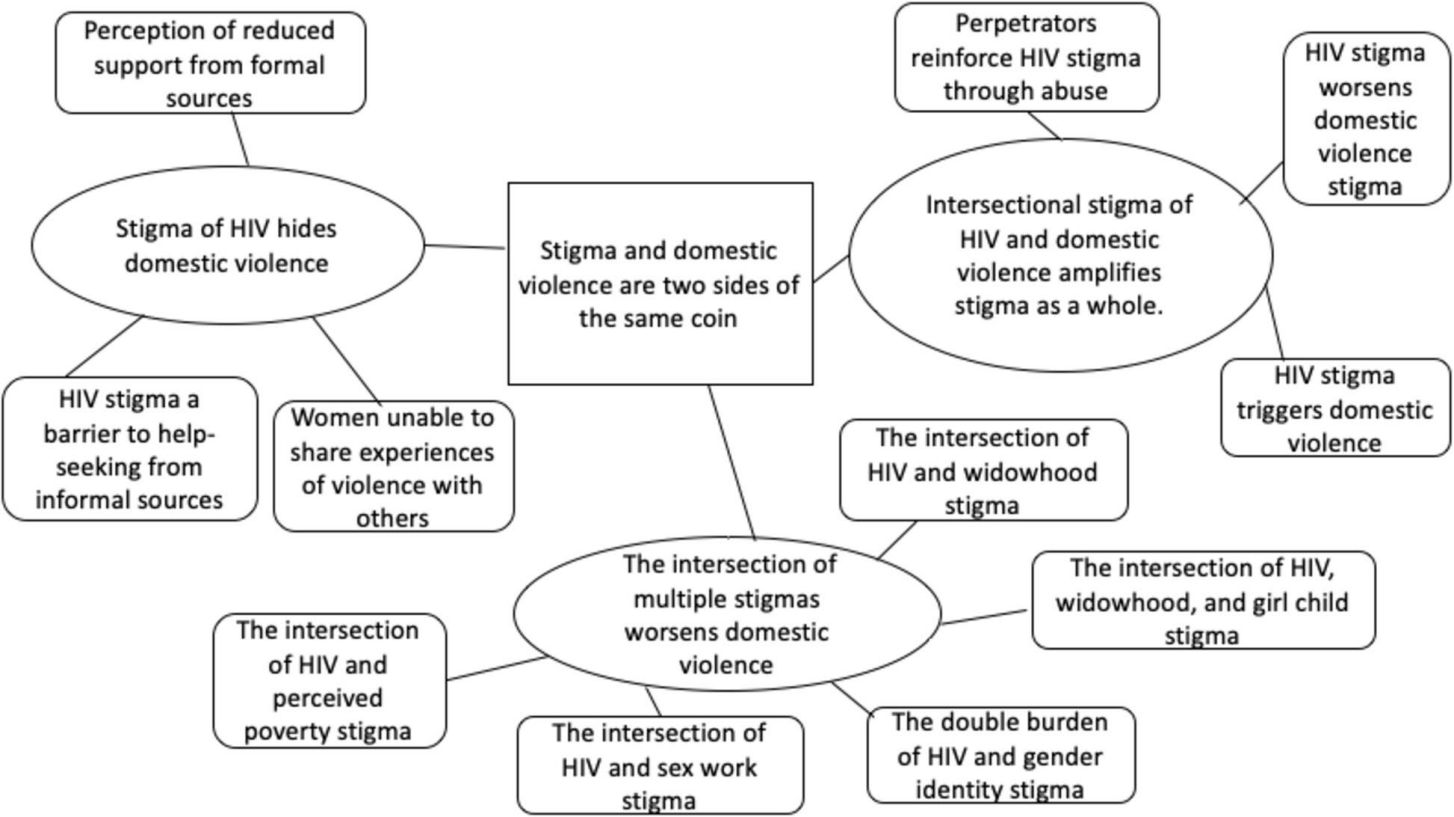
Development of themes using thematic network analysis. Boxes represent basic themes, ovals represent organizing themes, and center box is global theme

Interviews were conducted in Bengali or English and lasted from 30 min to over 2 h. Audio-recordings were transcribed and translated verbatim into English (by RM). De-identified transcripts were stored in an encrypted laptop and backup server. The methodological orientation underpinning the study was interpretive phenomenology as it describes experiences and perceptions of intersectional stigma for women living with HIV in this cultural context [32]. RM coded the data in Nvivo 12.6.1 qualitative software (QSR International, USA) using a mix of deductive and inductive coding (Additional file 4: Coding framework), so that both emergent themes and themes from the intersectional stigma framework were captured [33]. We used Thematic Network Analysis to describe patterns or themes in the data from different sources [34]: patterns from ‘basic themes’ were aggregated into broader ‘organizing themes’ and then abstracted into a ‘global theme’ that summarised the meaning within the dataset (Fig. 1). This analysis allows one to see how lower level themes (basic themes) come together and are explicitly connected to the higher order themes (organizing and global themes) (Fig. 1) [34]. Thematic network analysis is therefore appropriate for analyzing multilayered experiences such as intersectional stigma.

We remained reflexive throughout data collection and analysis by keeping detailed field notes, reflecting on the interviews, and making annotations and memos during coding. We considered how the interviewer’s positionality, in terms of ethnicity, gender, class, and HIV-negative status, in relation to the participants’ might influence the data collected and the interpretation of results.

### Interview safety considerations

Participants were informed that their participation was voluntary and would not affect the care they received at the NGO and that their data would be kept confidential. They were warned about potential risks of participation such as recounting painful memories. Interviews with women were conducted in accordance with the World Health Organization guidance on research on violence against women [35]. Specifically, the interviewer was trained to conduct interviews in a non-judgmental tone. Participants were offered breaks and the option of discontinuing the interview. The researcher checked for distress throughout the interview and spent time with each participant afterward to talk about positive aspects of their lives. Every effort was made to discuss potentially distressing topics in the middle of the interview and to always end on a positive note with questions on mental strength and hope. Although a distress protocol was available (stopping the interview and referring the participant to the NGO counselor or a nearby psychiatric facility), it was not needed for any of the interviews. All participants were followed up by the counselor, member of outreach staff, or researcher for up to a week after the interview to check for continued distress. Findings were shared with participants and the project partner. The researcher was trained in sensitive interviewing and had access to mental health and wellbeing services from UCL if she needed counseling for secondary trauma.

## Results

Women interviewed had been living with HIV for an average of 8 years and their median age was 35 years (Table 1). Most were Hindu, were from both rural and urban areas, all currently resided in West Bengal, and described themselves as being of low socioeconomic status. The majority of women were native Bengali speakers, while a small minority were native Hindi speakers (but spoke Bengali fluently) and mostly had families who had migrated from the neighboring state of Bihar. A diverse range of key informants were included, as described in Table 2. Women used the Bengali word “ghenna,” which directly translates to *disgust* or *repulsion* to denote stigma. They related their experiences of domestic violence to HIV stigma and how the violence itself was stigmatized and often worsened by their other marginalized identities.

Women talked about aspects of their identities and it was clear from the interviews that the intersection of multiple co-occurring stigmas shaped their experiences of stigma as a whole. Although the intersection of HIV and other stigmas is presented in distinct categories in this section, the findings should be interpreted as co-occurring when the results are considered as a whole [21]. Here we present the basic themes along with their overarching organizing themes: (i) the intersectional stigma of HIV and domestic violence amplifies stigma as a whole, (ii) the intersection of multiple stigmas worsens domestic violence, and (iii) the stigma of HIV hides domestic violence.

## Intersectional stigma of HIV and domestic violence amplifies stigma as a whole

### HIV stigma triggers domestic violence

Violence related to HIV stigma was pervasive. Most women interviewees had experienced domestic violence in their lives. Violence erupted for the first time after diagnosis or worsened for women who had previously experienced it. HIV stigma was a trigger for this domestic violence, with women reporting blame, isolation, physical abuse, and rejection from partners.

and in-laws. The different types of violence women experience are summarized in Table 3. Abandonment or being forced to move out of the marital home was one of the most severe forms of stigma-related violence reported.

“I was admitted [in hospital] for one and half months… then they discharged me, [my sister] brought me home, to my parental home, then I started calling my husband, he was like I’ll come today, I’ll come tomorrow, he kept saying that but he never came [Participant 001].

**Table 3:** Different forms of stigma-related violence experienced by women

	Types of HIV-related violence
Denial of medical care	Forceful interruption/suspicions around clinic visits
	Actively stopping (using physical violence) ART intake
	Tearing up all medical reports and denying having HIV
	Forced unsafe sex
	Not disclosing status to woman depriving her of treatment
	Preventing hospitalization even when very sick (woman needs to continue to do housework)
	No support with medical costs
	Emotional manipulation to stop ART intake
Neglect	No help with housework when sick
	Emotional neglect
	Woman having to care for herself when sick
Forced migration	Forceful eviction from shared household
	Women decide to leave on their own (fearing violence if family gets to know)
	Abandonment at hospital/women’s shelters (upon diagnosis)
	Forced to move out of village
Isolation	Forced to stay in one room of shared house
	No one speaks to woman and her children
	Specifically told not to attend social gatherings
	Utensils separated/not allowed to cook or touch family food
	Not allowed to use village pond
Nutritional deprivation/economic violence	Denied nutritious food
	Denied financial support (women have to start working for the first time in their lives)
	Denial of husband’s property
Publicizing status	Telling neighbours/extended family about woman’s status
	Gossiping about her status (behind her back)
	Utensils separated by in-laws at large gatherings
Intense psychological abuse	Verbal abuse about poor moral character
	Notions of untouchability reinforced through isolation/threats of denial of last rights
	Husband and in-laws gang up to blame the woman for “bringing HIV”
	Widows blamed for “killing husband”
	Women made to feel dirty (“smells bad”, “body riddled with worms”)

### Perpetrators reinforce stigma through abuse

Domestic violence itself played a role in reinforcing HIV stigma through verbal and emotional abuse. The most common form of domestic violence for women living with HIV was blame and ostracism. Blame for bringing the disease into the relationship can often reinforce stereotypes associated with HIV—poor moral character and an association with sex work—while ostracism reinforces stereotypes around untouchability due to fear of infection.

“.. if there are quarrels they [sisters-in-law] say sometimes, ‘you have such a disease you have no one to turn to except us, when you die then we only have to do everything, they will not touch you, other people will not touch you’... Even now they say such things, if there are the slightest quarrels” (Participant 030)

Reinforcing such stereotypes can in turn cause women to internalize the stigma. This has its own set of consequences, including poor help-seeking, withdrawal, and ultimately poor health.

### HIV stigma worsens domestic violence stigma

Domestic violence bears its own stigma as described earlier, but when a woman has HIV and faces domestic violence it is greater “because the stigma is not just on the domestic violence front, but it is also HIV stigma added on to it” (Official, domestic violence NGO). Women had the perception that others would be unsympathetic towards survivors of such violence because of their HIV status. There were reports of gossip among in-laws about HIV-related domestic violence. The intersectional stigma of HIV and domestic violence was distressing for women and caused them to withdraw socially.

“I don’t talk to anyone now, before I used to talk to everyone in the rented house [where in-laws are her neighbours], now I don’t talk to anyone… people gossip you see and I don’t like that (Participant 002).

## The intersection of multiple stigmas worsens domestic violence

### The intersection of HIV and widowhood stigma

Almost one-third of women interviewees were widows, as men were often diagnosed with AIDS and did not survive long. This particularly vulnerable group suffered from the intersectional stigma of widowhood and HIV. Widows suffered some of the worst violence immediately upon diagnosis. There were numerous examples, both from widowed women and from key informants, of how domestic violence from in-laws worsened as soon as the husband died:

“Those whose husbands die … they are tortured much more…I mean the family does not want to accept that their son is positive and so the woman also became positive, they will not accept since the man has died, the torture starts on the woman immediately…they turn around and claim that our son got infected from you” (Counselor).

The stigma of HIV was used by in-laws as an additional means of denying women their husband’s property by framing them as either immoral or infectious, such that they could not be allowed to stay in the marital home.

Then after my husband’s last rites were performed my younger brother-in-law, in order to stop me from staying [in the house], started disturbing me…‘I need the reports, you cannot stay in this house’… Then my sister-in-law said ‘she has this disease, if she doesn’t get tested then I will take the police to her [natal] house’ (Participant 013).

### The intersection of HIV, widowhood, and girl child stigma

In a patriarchal society such as India, birthing daughters is an additional source of stigma. Women who had given birth to daughters often spoke about the abuse they faced from husbands and in-laws, even when they did not have HIV. Stigma due to the lack of male children and widowhood increased women’s vulnerability to violence and eviction from the home.

“I had gone back there [marital home] once with the daughters, when my husband had died…So she [mother-in-law] was like, ‘go prostitute them and feed them, you also prostitute yourself, and when the girls grow up then get them into it as well. Go, go, go. Females, I will not keep females, it they were boys then at least they would have a place here, you don’t have boys, go, get lost.’ (Participant 008).

### The intersection of HIV and sex work stigma

Most of the interviewees who were sex workers were extremely reluctant to speak about their experiences of stigma: both related to HIV and their professions. However, they agreed that stigma would be greater if others found out about their HIV status and that there could be violence from clients if they found out: “Yes, he might [become verbally abusive] if he finds out he might” [Participant 026]. Their positive status meant that they were no longer accepted by the sex worker community in addition to never being accepted by the general population. One of the major difficulties faced by this group of women was their loss of jobs, which pushed them into poverty and stigma, this time at the hands of other sex workers:

What happens with sex workers is that they live in adjacent rooms, so [they think] ‘if the other sex worker knows I have HIV, she will use it to shoo away my clients, won’t let them come to me. She is HIV+, don’t go to her room, if you go then you will get it too’…so here the other woman tries to put the blame on her or put this mark on her…So for that reason she wants to conceal [her status]. So, there is this stigma within them, because this is their job and they may lose their job, and if they lose their income then other things will be a problem (Counselor).

### The double burden of HIV and gender identity stigma

Although we were unable to interview transwomen for this study, accounts from key informants who had worked with them shed light on their experiences of intersectional stigma. Unlike female sex workers whose sex work status might not be known, the transgender identity could not be concealed. The intersection of HIV and transgender stigma compounded the stigma experienced by transgender women because once they had HIV they were neither accepted by the HIV-negative transgender community nor by the HIV community:

“If am an individual belonging to “Other Sexuality” then I am stigmatized by society in different ways, and this is causing me to become isolated. But when I am within my peer group then [I feel accepted, but] when I am diagnosed with HIV, then I am stigmatized within my peer group, then where will I go now? A circle is being created within a circle, a box is being created within a box…when she is in that [trans] peer group she is being stigmatized [for being HIV positive] and when she is going to the HIV-positive group then she is being stigmatized for being trans, she is facing another round of stigma for being trans [and] positive. So, her place is much more vulnerable” (Public health official).

### The intersection of HIV and perceived poverty stigma

As socioeconomic position was not measured in quantitative terms, participants reported on their perceptions of poverty stigma. Women were specifically asked if poverty added to the stigma of HIV and almost all felt that poor women suffered more. A participant felt that the rich experience less abuse because people are generally afraid of them, whereas the poor need help, and this opens them up to further discrimination.

“Yes, it [discrimination] is more, see people talk about poor people more, no one can talk about rich people. People are afraid of them. [People] are not scared of poor people. They think at some point [the poor] have to come to us, but the rich think they don’t have to go to anyone. Because the rich do not need help” (Participant 019).

Another woman described how she felt the stigma of poverty intersected with that of HIV: poor people were thought of as dirty and bringing disease:

“They have money, no one will have *ghenna* towards them. They won’t. We don’t have money, they will say, ‘*dhur* who knows what she has, she can give it to us, let us stay away from her.’ That’s how it is” (Participant 014).

However, a small proportion of women felt that there was no difference in discrimination experienced by the poor and the rich and all suffered equally because of HIV stigma.

## Stigma of HIV hides domestic violence

### Women unable to share experience of violence with others

The first step in help-seeking is being able to tell others about the problem, but women talked about how they were unable to share their experiences of violence if it was related to HIV. Most were fearful of how others might react and some also felt ashamed of having HIV. HIV stigma-enforced silence on domestic violence caused women to suppress the pain within themselves:

“Want to say but it gets stuck. Lots of thoughts, what will they say or not say, if someone hears what will she do, will show *ghenna* or love” (Participant 023).

Despite domestic violence being stigmatized, women who had been experiencing violence before their diagnosis felt that they were able to talk to others about this regular violence from partners or in-laws, but felt compelled to exclude HIV from the conversation once they were diagnosed and the violence was worse.

“I had problems with him, I would have problems and leave for my natal home and stay there for months. Everyone would tell me ‘why do you leave like this’, I would be like ‘my husband gets drunk and verbally abuses me’, can I tell the neighbors that my husband has this disease, this would be buried within me” (Participant 027).

### HIV stigma a barrier to help-seeking from informal sources

HIV stigma was a barrier to help-seeking from friends and neighbors in matters of domestic violence. There were many ways in which this barrier worked. Firstly, women were afraid to ask for help due to fears of making their status public and thought it was better to bear the violence quietly:

“Yes, because of that fear [of revealing HIV status] I don’t want to tell [anyone about violence], that what will they think. The ones that want to help will help and those that don’t want to will not help. They will say a few words… I will have to listen to those taunts… people will say why does she have it, how did it happen…” (Participant 006).

Secondly, women believed that no one would help them because “she has HIV, the neighbors know, the fault will lie with the woman, it will be the woman’s fault. And everyone has *ghenna* towards this disease” (Participant 013). Thirdly, women felt ashamed to ask for help because they had HIV: “How will I ask from them? [laughs] How will I be able to tell them? I will be ashamed to even try to say anything” (Participant 012). A counselor from an HIV NGO who had seen many cases of domestic violence talked about how she had almost never seen a woman with HIV be helped by neighbors in cases of HIV-related family violence:

“Yes, she can say that, but how many people will come to save her is the question. Obviously, she will want to be saved, but how many people will come to her aid, I doubt if I can count the number on my fingers, I doubt anyone will come to her aid, if they know she is positive. In that case does anyone come to help? I don’t think anyone has ever come to help” (Counselor).

However, some respondents thought of the problem differently. They felt that if the woman was liked by the community in general she would definitely get help even if she had HIV.

### Perception of reduced support from formal sources

Despite half the women in the study experiencing violence after an HIV diagnosis, reporting to the police was rare. Reporting domestic violence to the police is considered an extreme step in India as it is considered a family matter. Women were mainly of the opinion that domestic violence should not be reported to the police if it could be avoided. Another problem with reporting to the police is that such a step can draw a crowd, which means her status would become public.

“Maybe they [police] will go and tell them [family] but then she will have to return to the same place, isn’t it? Maybe the police will go for 5 or 10 minutes or half an hour and tell them but the woman will never have happiness since now it [HIV status] has been made public … Now that it [HIV status] has been made public everyone will tell her to get lost, will have *ghenna* towards her. Every single person” (Participant 010).

Some women thought that structural discrimination within the law enforcement system meant that people living with HIV would not get help from the police. A sex worker who had reported violence to police and received help from them felt that “once they know [about HIV] will they [help]? They won’t” (Participant 024). Two women who had reported domestic violence to the police before their HIV diagnosis felt they would not have been able to do so had they known their status.

“I think they would have blamed me. ‘That the woman is a bitch her husband is good. This woman goes out of the house, works, brings sawdust, sells wood to eat, she must have messed around with a man.’ That is what they would say…” (Participant 011).

However, some respondents felt that women would get help from law enforcement if they tried to report domestic violence. A widow who went to the police after her remarriage got help from the police despite revealing her status to them.

Additional quotes supporting each theme have been added in Table 4.

**Table 4:** Themes with illustrative quotes

Themes	Subthemes	Illustrative quotes
Intersectional stigma of HIV and domestic violence amplifies stigma as a whole	HIV stigma triggers domestic violence	“There was a lot of trouble, lot of trouble [after diagnosis] …if one person quarrels and moves away, then another comes to quarrel, then after that one moves away, another one comes to quarrel. I had to run away to my mother’s house at night… there was trouble and quarrels… ‘where did you get it from, tell us who you had a relationship with’…” (Participant 019)
	Perpetrators reinforce stigma through abuse	What I have seen in the case of family violence, the projection that is there, ‘I got it from you’, … ‘you must have a relationship with someone, with whom you had sex, for which reason you have become positive, for which reason I have become positive’…then she has to face more abuse…I mean that reaches the level of physical assault (Clinical psychologist)
	HIV stigma worsens domestic violence stigma	“Yeah… if they have already been there …been experiencing, …domestic violence, and, yeah, there is no safe space…for them to talk. And similarly, … if they are diagnosed with HIV also, like it’s a kind of a … double stigma … for women. And they don’t talk about that [domestic violence] openly… even among women living with HIV, they don’t talk about domestic violence, you know. They only talk about HIV, that status, but often they don’t talk about the domestic violence part” (HIV Activist)
The intersection of multiple stigma worsen domestic violence	The intersection of HIV and widowhood stigma	“…When my husband died then they said that she walks on the bad road, that’s why she has this [HIV]…when I came to see the doctor here they said people thought I was really going somewhere else. When I came to see the doctor here I would not tell anyone” (Participant 014)
	The intersection of HIV, widowhood, and girl child stigma	“My father-in-law and mother-in-law… they threw me out… She [the widow] will live [in a corridor] by the entrance [to the house] with a [girl] child but they [neighbours] did not do anything… I stayed by the entrance for 6 months” (Participant 030)
	The intersection of HIV and sex work stigma	“…women are really neglected…because these are mostly trafficked women [sex workers] … maybe because of that the family rejects them more. A lot of families don’t want to take them back because she has been rescued in that way [from the sex trade]… ‘if we take her back our honor will be lost’. If they hear HIV positive then even more honor will be lost” (Counselor)
	The double burden of HIV and gender identity stigma	“…a transgender person is constantly stigmatized because of their gender. They are bullied…and when she is infected with HIV, then her stigma becomes a double stigma” (Public health official)
	The intersection of HIV and perceived poverty stigma	“Bigger [problem if people find out HIV status] for the poor. Because the rich will use their money to adjust it. It is a bigger problem for the poor. Problem from all sides. Living is very painful.” (Participant 023)
Stigma of HIV hides domestic violence	Women unable to share experiences of violence with others	“If he causes problems [violence] then I feel like the problems are crushing me from within…I sit alone quietly by that window… I stay quiet, don’t tell anyone. When there are problems within, I stay quiet at times and at times I [want to] let them [others] know that I have such problems. Then I think, ‘what’s the point in telling?’” (Participant 007)
	HIV stigma a barrier to help-seeking from informal sources	“No, [a woman] can’t [ask for her help for domestic violence]. No, she can’t, she can’t…she has HIV, the neighbors know, the fault will lie with the woman, it will be the woman’s fault. And everyone has ghenna towards this disease” (Participant 013)
	Perception of reduced support from formal sources	“Rather than help [after an episode of domestic violence] I think they [village representative] would have more ghenna towards me. More people would get to know than the few people who knew… That’s why I didn’t say these things. I just told them about the trouble [violence] but I didn’t tell them very much more” (Participant 19)

### Stigma amplification loop

**Fig. 2:**
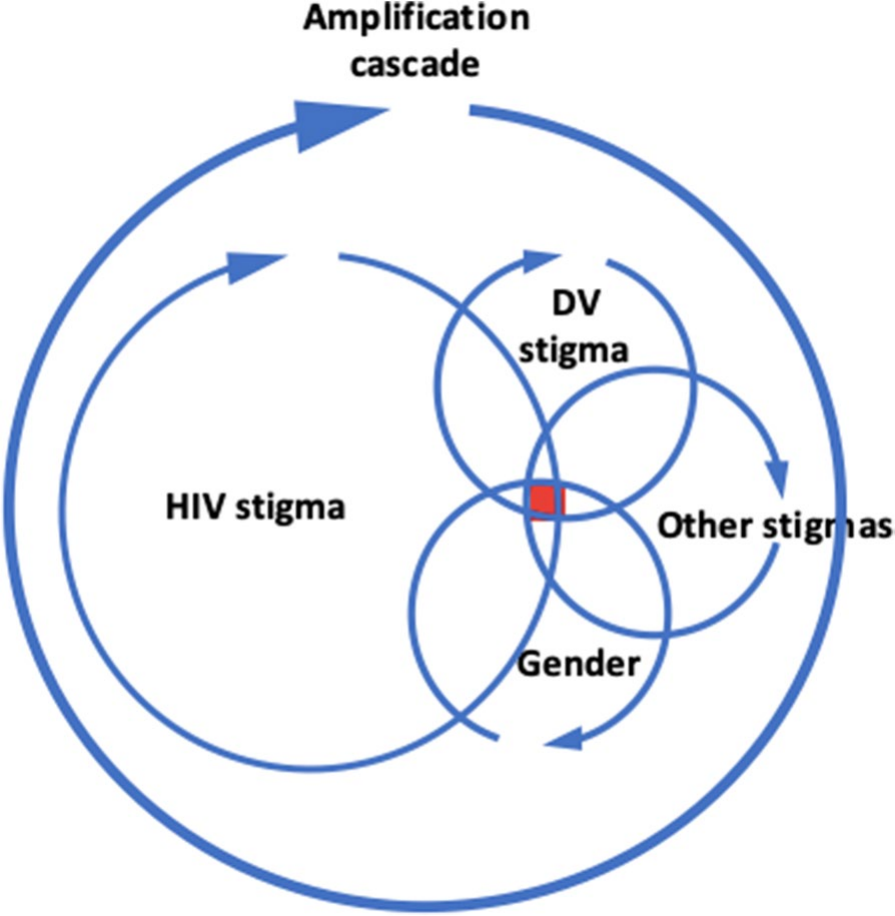
Stigma amplification loop for women with multiple marginalized identities

We have conceptualized our findings on the intersectional stigma of HIV, domestic violence, and other marginalized identities as a stigma amplification cascade (Fig. 2). Here multiple stigmas, such as those from widowhood, having a girl child, gender identity, engagement in sex work, and being a survivor of domestic violence, intersect to amplify the stigma experienced by women. This manifests as the increased violence evidenced in women’s accounts of their experiences after diagnosis. For example, a woman who experienced violence from her husband and in-laws before diagnosis was evicted from her household with her daughters once she was diagnosed and widowed due to HIV. Similarly, a sex worker who felt stigmatized due to her sex work status was now subject to verbal abuse from members of her own community because of the intersectional stigma of HIV and poverty (which was itself a result of her HIV status).

Our model therefore provides scope for recognizing that not only are women a homogenous group, but that the stigma of their other marginalized identities intersect with HIV stigma to shape each individual’s overall experience of stigma that manifests as violence.

## Discussion

It is well established that HIV increases the risk of domestic violence against women, but few studies have linked this violence specifically to HIV stigma [36–38]. Our findings suggest that the intersection of HIV stigma with that of other marginalized stigmatized identities amplifies the stigma as a whole experienced by women (Fig. 2). Specifically, domestic violence not only increases after an HIV diagnosis, but perpetrators use abuse to reinforce HIV stigma and HIV stigma worsens domestic violence stigma. The burden of violence is greater as women are unable to speak up about it to others or report it to formal sources. When women have additional marginalized identities, such as being a widow or a widow with daughters, the violence escalates further.

The recognition of multiple intersecting identities draws on intersectionality theory [17], which has been brought into the stigma literature to develop understanding of intersectional stigma; that is, the intersection of multiple marginalized or stigmatized identities or social positions shape a person’s overall experience of stigma [6, 20]. Some of these identities—gender, poverty, sex work, and gender identity stigma—were also uncovered in our study, but those such as widowhood and remarriage and having daughters emerged as prominent stigmatized identities in this setting. Although stigma related to caste and religion were mentioned, they were not prominent. Intersectional stigma experiences of inter-caste or inter-faith couples with HIV are a promising area for future research in this setting.

The theoretical literature has long recognized violence as an extreme form of enacted stigma (defined as acts of discrimination against the stigmatized individual) against people living with HIV [39–41]. The amplification of stigma as a result of women having multiple marginalized identities may therefore be seen as an extreme manifestation of intersectional stigma. While women’s experiences of HIV stigma-related violence may be viewed as a result of a confluence of various stigmatized identities (being a widow, having girl children, being a female sex worker, being a transwoman), the underlying role of gender discrimination in all these identities must be considered. This underscores the gendered nature of HIV stigma. For a woman living with HIV, stigma is always an intersectional experience in which her marginalization because of her gender is inextricably woven into her experiences of stigma due to HIV. This intersectional stigma often manifests as violence. Power imbalances within relationships have been cited as the main underlying causes of domestic violence [42]. Our findings show that HIV stigma reinforces and reproduces this power imbalance within relationships, which then manifests as worsened domestic violence against women living with HIV.

While other studies have highlighted the intersections of poverty and HIV stigma, none have related them specifically to HIV-related domestic violence [21, 43]. All the women who contributed to our study were of lower socioeconomic positions and felt that their experiences of stigma were magnified by being poor. Poverty exposed them to additional stigma and disempowered them such that it became harder for them to resist the violence they experienced. The interaction of stigma from multiple identities is complex and unpredictable, but our findings point to a synergy of multiple stigmas. Identifying additional vulnerabilities that result in increased violence against women living with HIV also underscores the need for additional stigma reduction interventions, social protection, and domestic violence services for them.

The intersectional stigma of HIV and domestic violence means that women are essentially unable to share experiences of violence with others or to report it to formal sources. Similar findings have been observed in other settings where women failed to report violence for fear of revealing their status [37, 44–46]. Unlike studies from Canada and South Africa, which found that stigma trapped women in abusive relationships [6, 13], Indian women found it hard to leave abusive relationships because of the social stigma of divorce, which was magnified by HIV stigma. This again is a function of gender norms that position women within the private sphere and as inferior to men. Collective action and community mobilization programs that bring women together to act as a group to dismantle structures of oppression, such as gender discrimination, can aid women’s fight against stigma-related domestic violence [47, 48].

People living with HIV are offered legal protection in India through the 2017 HIV/AIDS Prevention and Control Act. All women suffering from domestic violence are offered legal protection through the Protection of Women from Domestic Violence Act, of 2005. Public health services include free counseling and testing and free anti-retroviral therapy are offered to all people living with HIV. Additional benefits include a widow pension scheme, transport subsidies, and small monetary rewards for adherence to treatment [49].

The findings of our study may have implications for policy and practice. All women presenting at ART centers for treatment would benefit from being screened for domestic violence, considering that stigma often manifests as violence in their lives. Stigma reduction counseling should be strengthened at all ART centers, especially for women who report violence. Welfare benefits for widows might benefit from the provision of shelter homes, given the high rates of eviction faced by this multiply marginalized group.

Limitations of the study included women’s tendency to normalize experiences of violence but this was overcome by using trauma-informed, sensitive, and non-judgmental interviewing as far as possible. Although a diverse group was purposively sampled, the sample size did not allow for a full exploration of differences between groups. Women were asked open-ended questions about their multiple stigmatized statuses, but probing questions had to be added which may have led them to think of their identities additively rather than synergistically: a common problem with qualitative intersectionality research [50]. Finally, this qualitative study was conducted in one city in Eastern India (Kolkata) with residents from the state of West Bengal; findings cannot be generalized to all women living with HIV across India.

The strengths of the study included a diverse sample of women with multiple marginalized identities. Although it was conducted with an NGO in Kolkata, women visited from remote rural areas and we believe that we captured the perspectives of both rural and urban women. Key informant interviews with a diverse group of service providers were a valuable addition. It was not possible for the researcher to ask participants about every marginalized identity [50], but several identities emerged over the course of the interviews and added richness to the data. Finally, the study is the first to our knowledge to examine how multiple stigmas intersect to shape domestic violence experienced by Indian women living with HIV.

## Conclusions

The application of an intersectional lens on women’s experiences of stigma-related domestic violence highlights how multiple marginalized identities shape the life experiences of women living with HIV. Our findings point to several novel ways in which domestic violence may be reduced for these women: (1) domestic violence reduction interventions for women living with HIV must be accompanied by HIV stigma reduction interventions, and (2) women with additional marginalized identities must be prioritized for such interventions.

## Supplementary Information

Additional file 1. COREQ checklist.

Additional file 2. Participant interview guide.

Additional file 3. Key informant interview guide.

Additional file 4. Coding framework.

## Data Availability

Because of the sensitive nature of the data, and ethical restrictions, requests for access will be considered by Prof. David Osrin: d.osrin@ucl.ac.uk.

## Abbreviations

ART: Anti-retroviral therapy
DV: Domestic violence
HIV: Human immunodeficiency virus
NGO: Non-governmental organization
