# Supplementary material for: Women living with HIV face intersectional stigma from infection, domestic violence, and other marginalized identities: a qualitative study in West Bengal, India
Source: BMC Glob Public Health. 2025 Jan 10;3:4. doi: 10.1186/s44263-024-00122-w (PMC11724566; doi:10.1186/s44263-024-00122-w)
Supplement: Supplementary file 2 — Additional file 2. Participant interview guide. [file 44263_2024_122_MOESM2_ESM.docx]

| **Qualitative Interview Guide Related to HIV and Domestic Violence Stigma** | |
| --- | --- |
| **Theme** | **Interview Questions** |
| **Ice-breaker/intro** | 1. **Tell me more about your family? How many people are there in your family? How was it when you first got married? What is your family setup like?** |
| **Transition**  **Types and effects of stigma and discrimination**  **Enacted stigma**  **Perceived stigma** | 1. **Thank you for sharing that. Can you share with me the story of when you were first diagnosed with HIV?**   Probe:   - What were the initial signs and symptoms? - How did you feel when you were first told about your diagnosis?   **One experience that many people with HIV talk about are stigma (the negative ways that that people are viewed or feel because of their differences) and discrimination (being treated badly by others because of these differences). I would like to talk more about these topics now. I understand that these are sensitive topics, and some of these questions may bring to mind painful experiences. You are welcome to share only what you feel comfortable with, and can stop or take breaks at any time.**   1. **In what areas of life (e.g. health care, work, family, community gatherings) have you experienced stigma or discrimination related to having HIV? Has this (stigma) changed over the years?**   Probes:   - Were there other reasons you were discriminated against in that instance? (*probe: because of religion/caste, gender, poverty*) - Who was the discrimination coming from? - How did you feel at that time? - What did you do at that time? - How did you feel afterwards?  1. **Do you think your HIV status affects your children? Are they treated differently because their mother has HIV or maybe because they have HIV?**   Probes:   - is the mother only blamed? - Is the father blamed? - Are the children blamed? - Where does the blame come from? Family, neighbors, healthcare providers  1. **Let’s go back to your diagnosis once more. How do you think you may have acquired HIV? Do you think people are treated differently based on how they think a person acquired HIV?** (Probes: modes of transmission, sources of the stigma, how does this make you feel) 2. **What kinds of opinions or attitudes do you think people have about women living with HIV? What stereotypes do you hear about women living with HIV? (Or what assumptions do people make about women living with HIV?)**   Probes:   - What do people say about them as women? - What do people say about them as mothers? - What about their lifestyle? (substance use, sex life, etc.) - In your opinion, what is going through the minds of people who discriminate against women living with HIV?  1. **How about men living with HIV?**   Probes:   - Are men and women living with HIV viewed differently? In what ways? - Are they treated differently? In what ways?  1. **Let’s go back a bit further in time once more. Do you remember how you felt about people with HIV before you were diagnosed? How do you think those feelings impact the way you see yourself now?**   **People experience stigma and discrimination based on many other things besides HIV, including being poor, having experienced violence, being trafficked or engaging in sex work. Stigma may also differ based on how people think you were infected.**   1. **Can you describe any experiences you have with discrimination based on other parts of who you are? Are there some things about you / aspects of your identity that you think may cause some people to judge you negatively or treat you differently than others?**   Probes:   - Your religion/caste? - Your income level? - Your educational level? - Your age? - Your having children / not having children? - Your way of earning money? (e.g., sex work) - Having experienced violence (domestic/other sexual)  1. **How does facing more than one type of discrimination affect you? Affect other women that you know?** 2. **How do you think the negative ways you may have been viewed or treated by others because of your HIV status may impact your health?** (Probes: HIV care seeking, collecting/taking HIV medication, other health issues) 3. **Did you disclose your HIV status to anyone after you found out? Can you share stories of how people reacted when you told them?**   Probes:   - Reaction of your partner/in-laws/others (violence, abuse, support) - Reasons for non-disclosure  1. **Have you ever been beaten or humiliated by your husband or in-laws? Why do you think the violence happened? Why do you think men do this?**   Probes:   - Types and frequency of violence - Reasons for violence  1. **Did this (violence) change after you were diagnosed with HIV? What do you think might be some reasons for this change?**   Probes:   - Reasons for violence before and after HIV (gender norms, blame, stigma) - Difference in the types and frequency of violence (increase/decrease)  1. **Did you ever seek support or report these violent episodes? Has there been a change in where and who you seek help from since your HIV diagnosis?**   Probes:   - Source of formal/informal support/reporting - Reasons for seeking/not seeking help (HIV related/un-related reasons)  1. **How did people react when you reported the violence? Do you think being HIV+ makes it more difficult to seek help for domestic violence? Can you please explain why?**   Probes:   - Fear of revealing HIV status - HIV stigma  1. **How has this domestic violence impacted your adherence to HIV medications or your ability to go to the hospital for HIV care?** 2. **Today we have talked about many negative experiences that you have gone through. Can you tell me a little more about how this impacts your mental health?**   Probes:   - Sadness, stress, worry, hopelessness related to stigmatizing experiences - How this impacts medication adherence/physical health - How this impact other areas of life (childcare responsibilities, household chores, work if any)  1. **How have these feelings impacted your adherence to HIV medications or your ability to go to the hospital for HIV care?** 2. **Have you sought any help for the negative feelings that you have just described? Are you aware of services that are available that can help you with mental health issues?**   Probe:   - Mental health counselling services - Psychiatric services  1. **How are some ways you cope or manage some of these negative experiences? What gives you strength?**   Probes:   - Social support, friends/family - Support groups - Clinical support (doctors, therapists, social workers delivering HIV care) - Religion, spirituality - Relaxation - Focusing on work - Other  1. **Can you talk about the extent to which women with HIV that you know accept (or do not accept) themselves?**   Probes:   - Accept one another? - Support one another?  1. **How do you create/sustain friendships or other close relationships? How do you approach meeting new people?** 2. **(Put it at the start/ask screening Q) Are you aware of the new law that prohibits discrimination against people living with HIV/AIDS? Can you tell me a bit more about it?**   Probes:   - Do they plan to take action against acts of discrimination using this new law? If not, why?  1. **How would you like to see society change to become more accepting of women infected with HIV? How could these changes be accomplished?**   **Is there anything I have not asked you about that you think I should know about? (i.e., what have I missed in this conversation?)**    **Thank you very much for your time. Your responses will be very helpful for improving services for women living with HIV/AIDS.** |
| **Internalized stigma**  **Disclosure and**  **Anticipated stigma**  **Domestic Violence**  **Mental health and support**  **HIV/AIDS Prevention & Control Act 2017**  **Wrapping up** |  |
