# Supplementary material for: Women living with HIV face intersectional stigma from infection, domestic violence, and other marginalized identities: a qualitative study in West Bengal, India
Source: BMC Glob Public Health. 2025 Jan 10;3:4. doi: 10.1186/s44263-024-00122-w (PMC11724566; doi:10.1186/s44263-024-00122-w)
Supplement: Supplementary file 3 — Additional file 3. Key informant interview guide. [file 44263_2024_122_MOESM3_ESM.docx]

**Topic guide for Key Informant Interviews**

Thank you for taking part in this study. As you know, we want to understand the situation regarding stigma, discrimination and violence against women living with HIV (WLHIV) in Kolkata. At any time, you can call a stop to the interview, or say that you do not wish to answer a particular question. Do you have any questions before we start?

1. **Can you please tell me a bit about yourself? The work that you do?**
2. **How long have you been working with HIV+ patients? How has that been?**

**As we discussed, over the years HIV has changed from a killer disease to a more manageable infectious disease. Yet HIV stigma persists. It would be helpful if we could discuss this aspect of HIV, ie stigma and discrimination against people living with HIV/AIDS (PLHA), in greater depth in the following section.**

1. **Can you share with us some examples of areas where you see improvements and areas where things have remained the same?**

(Probes: clinical treatment, drug effects, opportunistic infections and other HIV related illnesses, HAART, emergence of VCTC centers offering free medication and counselling, life span, quality of life, stigma).

1. **How do you think the level of stigma towards PLHA is today compared to what you saw when you first started working with PLHA? What do you think might be some reasons for the change?** (Probe: awareness, acceptance of HIV)
2. **Can you share some stories/experiences that your patients/other PLHA getting treatment at healthcare facilities might typically face** (probes: during VCTC visit, during regular doctor visit, during ART collection, during planned surgeries in hospitals or emergency situations, during pregnancies)? [Adjust this question for appropriate interviewee eg faith leaders, teachers, social care workers, policy actors etc]
3. **What do you think causes people to stigmatize PLHA?** (Probes: moral judgement, blame, fear of contagion) **Do you think there is a difference in how PLHA are viewed or treated (especially in healthcare settings) if people know how they were infected?** (Probe: sexual routes, sex work, injection drug use, MSM, trafficking vs blood transfusions, spouses of bridge populations)
4. **Do you think that women with HIV are stigmatized differently than men? Why do you think so? In what ways is the stigma different?** (Probes: societal attitudes towards women, violence at home or elsewhere, moral judgment, association with sex work, infidelity).
5. **Do you think women disclose their HIV status to their partners or in-laws? Can you think of reasons for non-disclosure?** (Probe: violence, stigma) **Can you share some stories of how partners/in-laws reacted upon hearing of a woman’s positive diagnosis?** (Probe: Positive and negative reactions)
6. **Do you think violence is a problem in the lives of women with HIV?** (Probe: gender based violence, domestic violence). **Can you tell us a bit more why you think or do not think violence may be a problem for WLHA?** (Probes: traditional gender norms, blame, stigma).
7. **Do you think women who experience domestic violence are stigmatized?** (Probe: family affair, shame) **In what ways do you think that the stigma of domestic violence might be different for HIV+ women compared to other women?** (Probe: blame, HIV stigma)
8. **Do you think women living with HIV seek support or report domestic violence?** (Probe: friends, family, neighbors, NGO, police, healthcare providers, women’s commission) **Why do you think they do or do not report violence? Do you think the pattern of reporting (who they report to or seek help from) is different compared to women who do not have HIV? Can you think of any reasons for this difference?**
9. **Can you suggest ways in which violence reporting rates can be improved for HIV+ women?** (Probe: violence screening programmes at VCTC/ART centers, support services)
10. **How do you think violence impacts HIV+ women’s ability to access care or adherence to treatment?** (Probe: difficulty going for clinic appointments, taking medications on time, hiding medications from family, family not knowing HIV status)
11. **Are you aware of the HIV/AIDS Prevention and Control Act 2017? Can you tell us a bit more about it and how it works? Do you think the Act is actually working in terms of reducing discrimination against PLHA?**
12. **What do you think we can do to reduce the stigma against HIV+ people? How can the HIV/AIDS Prevention and Control Act be made more effective in your opinion?**
13. **How would you like to see society change to become more accepting of women infected with HIV? In what ways could these changes be accomplished?**

**Is there anything I have not asked you about that you think I should know about? (i.e., what have I missed in this conversation?)**

Thank you very much for your time. Your responses will be very helpful for improving services for women living with HIV/AIDS.
