# Supplementary material for: Women living with HIV face intersectional stigma from infection, domestic violence, and other marginalized identities: a qualitative study in West Bengal, India
Source: BMC Glob Public Health. 2025 Jan 10;3:4. doi: 10.1186/s44263-024-00122-w (PMC11724566; doi:10.1186/s44263-024-00122-w)
Supplement: Supplementary file 4 — Additional file 4. Coding framework. [file 44263_2024_122_MOESM4_ESM.docx]

| *1.HIV Stigma* |
| --- |
| 1.1 HIV stigma from natal family |
| 1.2 HIV stigma from partners_in-laws |
| 1.3 HIV stigma from neighbours, friends, others |
| 1.4 Anticipated stigma |
| 1.5 Perceived stigma |
| 1.6 Internalised stigma |
| 1.7 Reasons for HIV stigma |
| 1.8 No stigma experience |
| 1.9 How to change HIV stigma |
| *2. Non-HIV Related violence* |
| 2.1 Violence before diagnosis |
| 2.2 Violence after diagnosis but not related to HIV |
| 2.3 DV stigma |
| 2.4 Support for DV (pre-diagnosis) |
| *3. HIV Related Violence* |
| 3.1 Violence after diagnosis |
| 3.2 Violence_serodiscordance |
| 3.3 Violence_men diagnosed first |
| 3.4 Violence_women diagnosed first |
| 3.5 Reason for no stigma (violence)_ partners_in-laws |
| *4. Intersectional stigma* |
| 4.1 HIV and DV stigma |
| 4.2 HIV and girl child stigma |
| 4.3 HIV and widowhood stigma |
| 4.4 HIV and remarriage stigma |
| 4.5 HIV and religion stigma |
| 4.6 HIV and sex work stigma |
| 4.7 HIV and gender identity stigma |
| 4.8 HIV and rich vs poor stigma |
| 4.9 HIV and gender stigma |
| *5.0 Reduced help seeking_non-reporting* |
| 5.1 Reduced help seeking_formal |
| 5.2 Structural challenges |
| 5.3 Reduced help seeking_informal |
| 5.4 Hiding HIV related violence |
